# The IGF-II–Insulin Receptor Isoform-A Autocrine Signal in Cancer: *Actionable Perspectives*

**DOI:** 10.3390/cancers12020366

**Published:** 2020-02-05

**Authors:** Pierluigi Scalia, Antonio Giordano, Stephen J. Williams

**Affiliations:** 1 Sbarro Institute for Cancer Research and Molecular Medicine and Center for Biotechnology, Biology Department, Temple University, Philadelphia, PA 19122, USA; giordano@temple.edu (A.G.); sjwilliamspa@comcast.net (S.J.W.); 2 Istituto Somatogene per la Ricerca Onco-Genomica, ISOPROG, 93100 Caltanissetta, Italy; 3 Department of Medical Biotechnology, University of Siena, 53100 Siena, Italy; 4 Somatolink Foundation, Inc., Philadelphia, PA 19102, USA

**Keywords:** IGF(I/II/1R), Insulin-like Growth factor (1 or 2 or receptor), IR**^A^**/IR-A, insulin receptor isoform A, IGFBP, IGF binding protein, ITN, integrin, M6PR, mannose 6 phosphate receptor, TF, Transferrin, VTN, vitronectin, HIF, hypoxia-inducible factor, VHL, Von Hippel-Lindau gene product, OCT, off-context targeting

## Abstract

Insulin receptor overexpression is a common event in human cancer. Its overexpression is associated with a relative increase in the expression of its isoform A (IR**^A^**), a shorter variant lacking 11 aa in the extracellular domain, conferring high affinity for the binding of IGF-II along with added intracellular signaling specificity for this ligand. Since IGF-II is secreted by the vast majority of malignant solid cancers, where it establishes autocrine stimuli, the co-expression of IGF-II and IR**^A^** in cancer provides specific advantages such as apoptosis escape, growth, and proliferation to those cancers bearing such a co-expression pattern. However, little is known about the exact role of this autocrine ligand–receptor system in sustaining cancer malignant features such as angiogenesis, invasion, and metastasis. The recent finding that the overexpression of angiogenic receptor kinase EphB4 along with VEGF-A is tightly dependent on the IGF-II/IR**^A^** autocrine system independently of IGFIR provided new perspectives for all malignant IGF2omas (those aggressive solid cancers secreting IGF-II). The present review provides an updated view of the IGF system in cancer, focusing on the biology of the autocrine IGF-II/IR**^A^** ligand–receptor axis and supporting its underscored role as a malignant-switch checkpoint target.

## 1. The Insulin–IGF Ligand and Receptor System in Cancer

**Figure 1:**
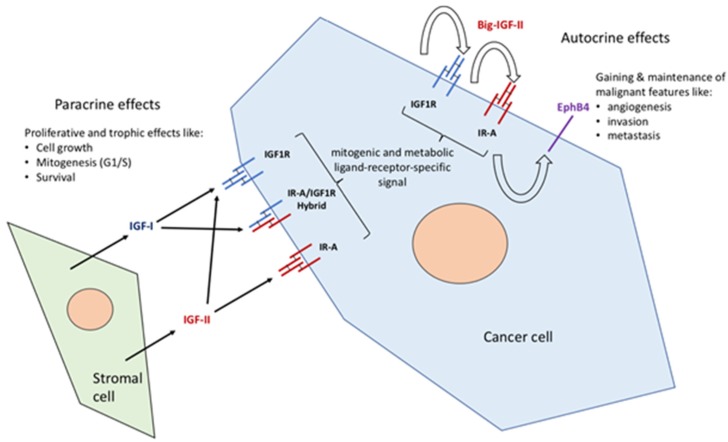
The family of insulin/IGF ligands and receptors in cancer: an updated functional overview. New contextual evidence points at a differential rather than overlapping role of IGFs and their TK receptors. In particular, the role of IGF-II and its cancer-secreted variant (Big-IGF2), as the most commonly expressed IGF ligand in malignant cancer cells, along with the A variant (exon 11) of the insulin receptor (IR**^A^**), an almost ubiquitously expressed variant of the IR in cancer binding IGF-II (and its high molecular form expressed in cancer) with high affinity, has gained additional interest on the basis of its ligand-receptor-specific (and IGF1R-independent) ability to tightly control the protein expression of an angiogenic invasion metastatic factor such as EphB4 [14]. This type of effect fully differentiates the outcome and relevance of the autocrine IGF-II/IR**^A^** signal towards gaining and/or maintaining malignant features. It also allows new anti-target scenarios within the IGF family to be envisioned and provides a mechanistic explanation for the observed failure of single therapy blockade of the IGF-I receptor in clinical trials.

The family of the insulin and IGF ligands and receptors are known for their central metabolic and growth-related functions spanning throughout phylogenetically distant organisms [1,2]. Up to the late 90s, the working model for the role of insulin, IGFs, and their receptors in cancer was based on a scenario dominated by two cousin receptors (the IGF-IR and the insulin receptor) used by their own ligands (IGF-I for the IGF-I receptor and insulin for the insulin receptor), with the IGF-I receptor being considered the sole active mediator of the IGF-I and IGF-II effects, making the latter a favorite target for halting the actions of IGFs in cancer [3,4]. This paradigm (the IGF1R mandatory transducer hypothesis) has undergone many changes over time with the realization, first, that the IGFIR is able to form hybrid variants with the insulin receptor [5,6] and, second, that the insulin receptor (IR) could mediate IGF-specific effects. Indeed, genetic evidence of the permissive role of the insulin receptor in a number of developmental and body-size effects mediated by IGF-II had been shown in genetic studies conducted both in null mice [7] and in transgenic mouse models [8]. However, cellular studies were not able to reproduce such a result in vitro until a specific isoform of the insulin receptor, lacking 12 aa in the extracellular portion corresponding to exon 11 (IR**^A^**), was shown to be the high-affinity receptor for IGF-II in both fetal and cancer cells [9]. This finding, besides changing a long-rooted view, also presented a distinct role for the insulin receptor far beyond defining it as a pure metabolic and growth permissive mediator. A number of subsequent studies have also demonstrated insulin and IGF ligand-specific differences in their activation of the IR**^A^**. In particular, such differences have been demonstrated at the gene expression level [10] and at the signaling level [11,12]. In this regard, it is worth noting that IGF-II has been found to be able to bind and transduce signals via both the homo-tetrameric, high-affinity RTKs (IGF1R and IR**^A^**) and via its hetero-tetrameric (IGF1R/IR**^A^**) hybrid receptor in cancer [13]. This amplifies the range of cancer-promoting autocrine signals exerted by IGF-II at the cellular level. The ultimate demonstration of the potential of the autocrine IGF-II/IR**^A^** axis in cancer came from the recent finding that, when activated by IGF-II, the IR**^A^** variant is able to acutely and reversibly activate a post-translational, ubiquitin-dependent, and IGF1R-independent degradation rescue signal, causing EphB4 ectopic expression in malignant mesothelioma cell lines [14]. Altogether, these reports fully disprove the old concept of purely redundant biological roles between IGF ligands and RTKs, and point at their contextual co-expression patterns as key indicators of the predominant and/or parallel effects exerted in cancer cells according to the pre-existing permissive or inhibiting signaling network. These contextual ligand–receptor interactions exerted by autocrine IGF-II and the contributions of paracrine IGF signals in cancer are summarized in Figure 1.

## 2. IGF-II is a Bona Fide Oncogenic Ligand Tightly Regulated Under Development and a Commonly Selected Self-Stimulatory Signal in Cancer

**Figure 2:**
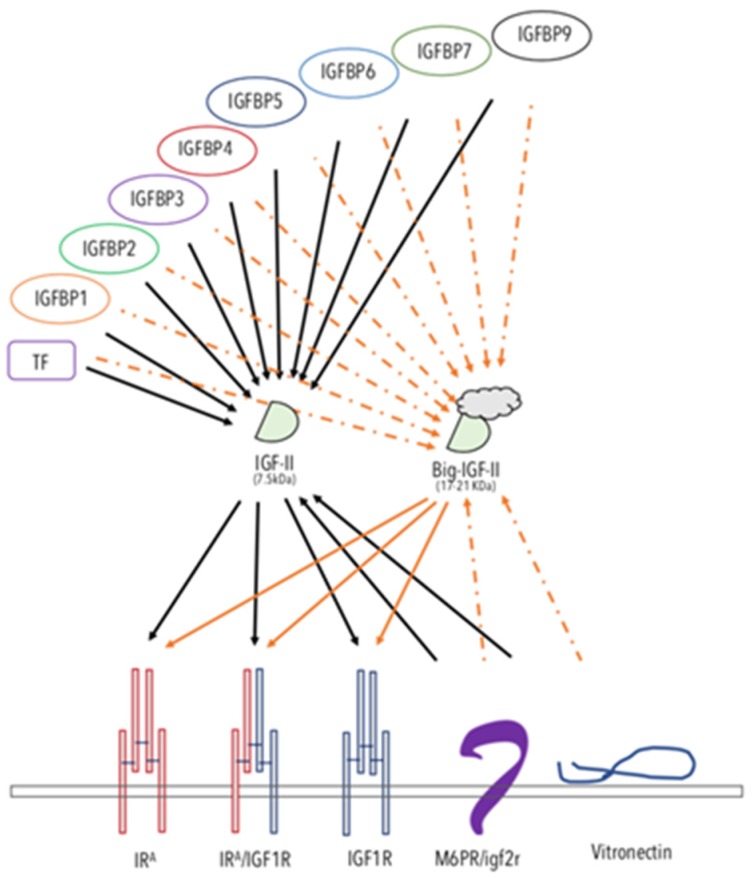
The IGF-II-binding/-neutralizing and -transducing system. The schematic figure summarizes the interactions reported in the literature for IGF-II. The known soluble, extracellular and/or membrane-bound IGF-II-binding proteins are displayed. The solid arrows represent experimentally supported interactions. The dashed arrows represent interactions that have been shown to either be impaired or not yet experimentally confirmed. Arrows from a ligand to its RTKs indicate activating–transducing properties. Arrows towards IGF-II indicate a binding–neutralizing effect. The overview of the comprehensive IGF ligands system role in cancer is shown in Figure 2.

In comparison to IGF-I (the main growth-hormone-induced ligand and physiological effect mediator) and as discussed below, IGF-II undergoes different and extensive regulation at the genetic, epigenetic, and post-transcriptional levels. Interestingly, the escape from such tight regulation, as observed in cancer, offers IGF-II distinctive advantages over IGF-I, mainly linked to its ability to activate specific developmental, cellular, and cancer-promoting signals via Insulin receptor A. Overall, IGF-II (a) has a wider possibility of transcriptional regulation and control at the gene promoter level via its four promoters and 10 exons [15,16], all of which produce a pre-pro-hormone and four isoform variants; (b) is epigenetically regulated via DNA-methylation-dependent and -methylation-independent mechanisms [17,18,19,20,21,22,23] with a paternal-restricted expression pattern which is typically lost in cancer (loss of imprinting), causing increased/biallelic expression and bloodstream secretion levels [24,25,26,27,28,29,30]; (c) displays post-translational variants derived via differential processing of its pre-pro-hormone leading, to an O-glycosylated high molecular weight form (also known as Big-IGF2) [31,32] retaining its binding and signaling activity for IR**^A^** [33] but with acquired capability to elude physiological binders such as the high-affinity scavenging receptor also known as igf2R (binding mannose 6-phosphate as well) and IGFBP3 [34]. This and the potential clinical implications have also been reviewed in References [35,36]. Finally, (d) additional types of regulation of the igf2 transcript linked to non-coding RNA products have also been demonstrated, adding a layer of additional regulation for the igf2 gene [37,38,39,40,41,42,43]. The escape from any of these regulatory mechanisms make IGF-II and, more so, its cancer-secreted variant (Big)IGF-II, an ideal autocrine signal for highly demanding cellular requirements such as those found throughout the tumorigenic process [33,34,44]. No less important, (e) IGF-II binds to the IGF1R, the Insulin receptor isoform A, and their hybrid tetra-dimeric forms under different physiological and pathological contexts to exert ligand-receptor-specific cellular effects [7,9,14,45]. The contextual roles mentioned above for IGF-I and IGF-II ligands in cancer are graphically summarized in Figure 2.

## 3. The IGF-II Binders: A Fine-Tuned System for the Control of IGF-II Levels in the Extracellular and Tumor Microenvironment 

*Igf2R/m6pR.* The non-transducing/scavenger high-affinity-binding membrane-bound protein known as igf2 receptor (reviewed in Reference [46]), initially thought to be an IGF-II biological mediator, exerts, indeed, most of its IGF-related effects by neutralizing IGF-II and subtracting it from other transducing interactions (namely from the IR**^A^** and the IGF1R receptor tyrosine kinases). The key evidence for such a view comes from the demonstration of the absence of a TK domain in its cloned structure [47] and from the oncogenic effect shown by null mutation of igf2rR/m6pR in mice [48]. Indeed, the tumor-suppressing effect of the igf2R/m6pR can be interpreted as further demonstration of the oncogenic potential of IGF-II when present in high levels in vertebrates either at focal tissue levels and/or in the whole organism bloodstream.

*The IGFBPs 1-7 and 9.* Insulin-like growth factor-II has been shown to bind to most of the soluble extracellular proteins of the IGFBP family, as reviewed elsewhere [49,50,51]. The cumulative effect of IGF-II binding proteins towards the IGF-II levels in the bloodstream might mitigate its increased exposure to local tissues. As a result, some authors have to proposed the use of recombinant fragments of IGFBPs as tools to counteract IGF-II oncogenicity. However, the fact that cancer-secreted IGF-II has been found to interact poorly with IGFBPs [34,52,53] might be seen as an escape mechanism for all those cancers using IGF-II as an autocrine growth factor to sustain/maintain their malignant growth features. These potential limits should be taken into consideration. 

*Transferrin (TF).* TF has been shown to be a constitutive component of the 150kDa trimeric IGF binding protein complex found in the bloodstream [51]. Its binding to IGFs (I and II) is less strong than other IGF–IGFBP interactions (where the highest affinity is shown with IGFBP3), and its physiological role is still to be determined.

*Vitronectin (VTN)*. VTN is a constitutive component of the extracellular matrix, involved in cell-to-cell interactions [54,55]. VTN has been known to bind integrin (ITN) alpha5beta3 and, as such, has been also referred as to integrin receptor [56]. Interestingly, VTN, which bears a somatomedin-like domain, binds IGF-II with high affinity [57,58]. Although the physiological and pathological roles of VTN interaction with IGF-II are still to be determined, some evidence points at a suppressing role of VTN on IGF-II-induced proliferation and migration via interference with the IGF-II mitogenic signaling (Scalia et al., manuscript in preparation). 

Overall, the studies on IGF-II physiological binders are in agreement with the genetic studies supporting a distinctive cancer-promoting role for this IGF, differentiating it from its related cousin, IGF-1. The finding that cancer-secreted IGF-II (big-IGF-II) skips the binding control exerted on mature IGF-II by the IGFBPs (as graphically summarized in Figure 2) suggests that more specific targeting strategies should be considered in order to target this factor in its cancer-specific context.

## 4. Autocrine IGFII and the IR**^A^** Isoform Co-Expression in Cancer: At the Root of IGF-I Receptor Block Resistance 

A number of historical results obtained in igf1r null murine fibroblasts (r-cells) both in absence or presence (r+) of human IGFIR expression abundantly demonstrated the isolated mitogenic and growth-linked effects of the IGF-I receptor as a key permissive signal for most of the non-IGF RTKs already targeted in therapy [59,60]. This triggered the development of a number of IGF-IR specific MAbs [61,62,63,64] and small molecules [65,66]) by the pharma industry in the first decade of the new millennium [67]. Although the experimental evidence showing a functional role for the IGF-II/IR**^A^** both in embryonal fibroblasts and in cancer has been available since the late 90a, these findings did not seem to affect the rush of drug developers to bring IGF-IR specific blockers to clinical trials. The specific single blocking of IGFIR in phase II clinical studies failed [68,69]; the extent of the negative impact of anti-IGF1R monotherapy drugs in clinical studies because of the underscoring of the IGF-II/IR**^A^** role could have been easily avoided by including IGF-II/IR**^A^** testing in the associated companion diagnostics required for the selection of responsive patients [70]. Interestingly, in 2006, a human anti-IGF-II MAb with pan-IGF-II blocking capabilities (neutralizing both the mature and high molecular weight IGF-II variant) was developed [71] as an alternative blocking strategy to IGFIR [72], clearly underestimating the advantages and the differential biological roles of the two therapeutic approaches. Unfortunately, the IGFIR blockers results have delayed the opportunity to test the effectiveness of molecular anti-IGF system targets based upon existing and new evidence revisiting the “IGF1R mandatory transducer hypothesis”. This trend should be reversed based on the cumulative validated biological knowledge of this system (with regards to the IGF-II/IR**^A^** axis role), which is supported by mechanistic explanation and scientific strength, ultimately disclosing the biological differences among IGF ligands and their TK receptors (IR**^A^**, IGF1R, and their hybrid variants) in mediating specific and contextual cancer-actionable effects that are now exploitable in the genomic-driven age [9,11,13].

## 5. The Autocrine IGF-II/IR**^A^** System and the Malignant Switch in Solid Tumors: Hints from the Hypoxic Network 

Hypoxia is an intrinsic feature of solid cancers’ tridimensional growth, affecting the inner core of the growing tumor tissue at the pre-vascular stage and clearly affecting the extracellular tumoral microenvironment. Under these circumstances, a tight sequential relationship is established between hypoxia and the expression of hypoxia-induced genes, in which HIF isoforms and VHL have been shown to play a major role [73,74]. Among the factors that have been shown to be induced or upregulated under hypoxic conditions are VEGF, EphB4, and IGF-II [75,76,77,78,79,80,81,82,83]. However, if for VEGF and EphB4 a solid base of supporting evidence has established their role in angiogenesis and cancer blood vessel formation, in the case of IGF-II, its angiogenic role in the literature has been variably and interchangeably associated with the angiogenic role of IGF-I. Indeed, as mentioned before, there is evidence supporting the notion that IGF-I and IGF-II are all but interchangeable molecules under both physiological and pathological conditions, as shown by their differential affinity and signaling properties via the known IGF1R/IR RTKs. All these structural and ligand–receptor interaction differences provide plenty of biological opportunity for their diversified use by the cell under hypoxic conditions (typical of early-stage and overtly malignant cancers). As for the association of IGF-II with the hypoxic tumor microenvironment, what we know from the published literature is that (1) IGF-II, but not IGF-I, is responsible for the hypoglycemic paraneoplastic effects observed in a number of patients affected by aggressive solid cancers (IGF2omas) [35,36]; (2) that IGF-II (as well as VEGF-A and EphB4) can be upregulated by HIF and hypoxia [83,84,85]; (3) that VEGF, which also exerts autocrine signals [86], can be upregulated via the IGF-IR and the IR**^A^** [87,88,89] and is under the control of IGF-II and its autocrine loop under hypoxic experimental conditions [14,84,90]. Interestingly, (4) Hypoxia-induced HIF2alpha can regulate IGF-II [91] and (5) IGF-II can upregulate HIF1alpha, which is an inducer of VEGF [14,90]. All these functional links observed in solid cancers can determine a number of coordinated local events towards the acquisition and/or maintenance of angiogenic, invasive, and metastatic potential, and are compatible with an underscored role of IGF-II in the angiogenic switch, supporting its validation as an anti-angiogenic target. It is worth noting the effects that autocrine IGF-II exerts exclusively via the IR**^A^** independently of the IGF1R, such as in regards to EphB4 acute protein level regulation in certain cancers such as malignant mesothelioma [14], making the IGF-II/IR**^A^** signal in these cancers a distinctive, non-redundant ligand–receptor loop with targetable value. This has been observed in vitro and ex vivo using cancer cells exposed to their conditioned media (pH ~6.9–7.2), a feature common to the extracellular conditions found in solid cancer microenvironments in vivo.

## 6. Learning from the IGF System Targeting in Cancer: Not All Ligand–Receptor Interactions are Created Equal (Context is “All You Need”) 

As in a chess game, any winning move or strategy comes from failure. In the case of the realization of the importance of the IGF system in cancer, the biological relevance of some of its family components, namely the IGF1R as the supposed sole mediator of the IGF-I and IGF-II effects, came early in the drug industry game, although experimental data related to atypical variants [92,93] and hybrid receptors with its related cousin, the insulin receptor (IR) [94,95,96], were already known. Indeed, the overexpression of the IR in cancer, first reported in breast tumors and related cell lines [97,98], has not been interpreted as relevant by the supporters of the “IGF1R mandatory-transducer” hypothesis, based on the assumption that the IR serves solely as a purely metabolic transducer, of which overexpression in cancer cells merely provides metabolic advantages over normal tissues with regards to nutrient consumption [12]. This scenario, reproducible but incomplete, began to be revealed as a fallacy when the short isoform of the IR (not expressing its exon 11 on the extracellular domain) was shown to be an onco-fetal high-IGF-II-affinity receptor. In this context, the failure of the clinical trials (and the many compounds which have not passed the preclinical stage) for all those drugs directed against the IGF1R (reviewed in Table 1A,B) did not come as a surprise, in contrast to the main supporters of its direct targeting [68]. Even the strategy of a double block of the IGF1R and the IR would have the same limits (and counter-effects) as shown by the clinical trials of linsitinib [99,100,101,102,103], a small molecule inhibiting both RTK receptors. This led to a recent approach conveyed in the development of xentuzumab and disigitumab (double anti-IGF1/IGF2 MAbs), targeting the known IGF ligands rather than their RTKs [104,105,106], currently in phase I testing. Although this approach (that of targeting the IGF ligands rather than their RTKs) may gain more leverage in phase II trials, the risk at phase III remains, since the growth-related, trophic, and protective effects of IGF-I on muscle tissue, bone, and other organs’ cellular components (such as the physiological stem cell compartments) may induce systemic effects opposite to those intended (especially in pediatric patients). These observed and potential off-target effects linked to the targeting of the individual as well as combined IGF family components can be explained in terms of “off-context targeting” (“OCT” effect), since each of these ligands and receptors plays a central role in physiological growth and metabolism from the embryonal stage to adulthood. An exploitable alternative to the approaches reviewed herein is the targeting of their known cancer variants in their pathological context. This could include, for example, the targeting of a context-selected IGF/RTK complex with agents discriminating their individual physiological components from their pathological variants. Specifically, the view supported by our groups and others [9,14,45,107,108] that the autocrine IGF-II/IR**^A^** ligand–receptor complex in cancer bears the required biological relevance and contextual pathological value would justify such a therapeutic strategy. Contextual conditions that would benefit by this ligand–receptor targeting are further discussed below.

## 7. Targeting the Autocrine IGFII/IR**^A^** Loop in Cancer: A Further Treatment Co-Target for Current Checkpoint Therapies? 

It has become increasingly evident how tumor-microenvironment-linked autocrine ligand–receptorial systems can play a distinctive biological role in escaping the effects of targeted monotherapies [157,158,159,160]. For example, VEGF-A and ADAM9 have been linked to resistance to dabrafenib as a result of downregulation of miR-126-3p [161]. This type of resistance could share autocrine IGF-II/IR**^A^** block sensitivity, these factors being actual (VEGF-A) or potential (ADAM-9) autocrine IGF-II-signaling-regulated targets. In other cases, IGF-II has been found to be directly responsible for the acquired resistance to targeted drugs [162]. Other studies have highlighted the relationship between RTKs and resistance to immune checkpoint inhibitors [163,164]. Even though the concept that an increase in RTK ligands acting in a paracrine or autocrine manner is responsible for native and/or acquired drug resistance in not new [165], no study so far has directly addressed the role of autocrine IGF-II through its RTKs (IR**^A^** and IGFIR) under these circumstances. Altogether, the evidence that IGF-II is also used by cancer stem cells, besides being commonly expressed in solid cancers [166,167,168,169], supported by the findings already obtained using genetic, molecular, and cellular approaches, suggests further new hypotheses. In particular, the observation that the presence of autocrine IGF-II loops is associated with overtly malignant cancer cell lines [14,45,94,170,171,172,173] and that IGF-II over-expression overlaps with those cancer types currently poorly responsive to immune checkpoint therapy (such as malignant mesothelioma [174,175,176], glioblastoma [177,178], and pancreatic carcinoma [179,180]) along with certain BRAF-inhibitor-treated recurring cancers [181,182] suggest that the autocrine IGF-II/IR**^A^** axis role under these circumstances should be investigated and its targeting potential experimentally vetted. Importantly, a sufficient amount of scientific evidence differentiating the biological and contextual pathological roles of the two IGF-II receptor tyrosine kinase signal transducers, namely the IGFIR and the IR**^A^**, has been produced to clear out the doubts and unmet past expectations linked to the failed strategy of IGFIR blocking [68,69]. In summary, the autocrine IGF-II/IR**^A^** ligand–receptor axis plays a key role in mediating IGF cancer-promoting effects, which have only recently begun to be revealed in terms of their distinctive and IGF-IR-independent signaling patterns. Specific preclinical studies are needed to address the full potential of this self-stimulatory axis and its specific molecular regulatory network in current precision oncology strategies. In particular, regarding the autocrine circuits, the receptorial machinery, and the signaling network active under hypoxic conditions, and potentially conditioning the benign vs malignant phenotypic switch, it is worth noting the recent demonstration of the receptor-specific effect that the IGF-II autocrine loop exerts via the IR**^A^** in regards to the acute protein level regulation of the angiogenic kinase EphB4 [14]. This was observed in vitro using cancer-cell-conditioned media (pH ~6.9–7.2), this hypoxia with low pH being a common feature to the extracellular conditions found in solid cancer microenvironments in vivo. 

## 8. Conclusions and Future Perspectives 

Cumulative evidence on the autocrine IGF-II biology, further strengthened by our recent findings on the role of the IGF-II/IR**^A^** signal [14], support a growing targetable value of this cancer-secreted ligand in order to block or revert its effects during malignant progression in a variety of solid cancers. In summary, the autocrine IGF-II/IR**^A^** ligand–receptor axis plays a key role in mediating IGF cancer-promoting effects, which have only recently begun to be revealed in terms of their distinctive and IGF1R-independent signaling patterns. Selective targeting strategies and new translational studies aiming to pinpoint patient response and validate the therapeutic value of cancer-secreted IGF-II and its non-overlapping RTK effects (such the IGF-II/IR**^A^**-dependent expression of EphB4 in malignant mesothelioma [14]) will prove essential in positioning the block of this established cancer-driving axis among the already validated precision oncology therapeutic agents. Specific preclinical studies are needed to weigh the full potential of this self-stimulatory axis and its specific molecular regulatory network in current personalized strategies.

## Figures and Tables

**Table:** 

(A)			
Small Molecules			
IGF Targeting Drug Type/Name	Malignancy	Clinical Phase Achieved	Refs.
IGF1R specific TK inhibitor(s)			
BMS-754807	Solid tumors	I	[109]
	hormone resist. breast cancer	II	[110]
KW-2450	advanced solid	I	[111,112]
IGF1R/IR dual TK inhibitor			
Linsitinib (OSI-906)	Solid tumors	I	[99,100,101]
	Adrenal Carc.	III	[101,102]
	colorectal	I	[100,103]
Immunological approaches for present (NK-mediated) and foreseeable (T-Cell-mediated) targeting of the IGF-system			
First generation target Rx (single IGF targeting Mabs proposed as monotherapy):			
MAbs anti-IGF1R			
Dalotuzumab (MK-0646)	Solid tumors	I	[113,114] [115] [116] [117]
	Neuroendocrine	I	
	Colorectal	I	
	SCLC	I	
	NSCLC	I/II	
Figitumumab (CP-751871)	Sarcoma	I	[118]
	Solid tumors	I	[119]
	Adren. Carc.	I	[120]
	Ewing	I/II	[118]
	Prostate	II	[121,122]
	Colorectal	II	[123]
	NSCLC ^a^	I/II/III	[124,125,126]
	Mult. myeloma	I	[127]
Ganitumab (AMG-479)	Solid tumor	II	[128,129]
	Pancreatic	I, II, III	[130,131,132]
	Ewing	II	[133]
	breast	II	[134]
	colorectal	II	[135]
Cixutumumab (IMC-A12)	hepatic	I/II	[136]
	pancreas	I	[137]
	thymus	II	[138]
Robatumumab (MK-7454)	sarcoma	II	[139]
	colorectal	II	[140]
Istiratumab (MM-141)	pancreatic	II	[141,142]
R1507	solid tumor	I	[143]
Second generation target Rx (multiple RTKs or ligands targeting MAbs)			
MAbs co-targeting of IGF1-IGF2 ligands			
Xentuzumab(BI-836845)	NSCLC	I	[104]
Dusigitumab (MEDI-573)	solid tumors	I	[105,106]

**Table:** 

(B)				
Small Molecules				
IGF targeting Drug Type/Name	Tumor Models Tested	Preclinical Assessment	Clinical?	Refs.
IGF1R specific TK inhibitor(s)				
NVP-AEW541	Multiple myeloma	In vitro	No	[144]
	Musculoskeletal, Ewings	In vitro, xenografts		[145]
	fibrosarcoma	In vitro, xenografts		[146]
Tyrphostin AG-1024	breast cancer cells osteosarcoma cell lines pancreatic cancer cell lines	In vitro In vitro	No	[147] [148] [149]
BMS-536924	ovarian cancer cell lines	Increases radiosensistivity		[150]
IGF1R/IR dual TK inhibitor				
AZ12253801	NSCLC	In vitro cytotoxicity, soft agar	No	[151]
	Colon adenoma	APC min +/− mouse model		[152]
LL28	Lung cancer	In vitro cytotoxicity, xenograft, KRAS lung murine model	No	[153]
Immunological approaches for present (NK-mediated) and foreseeable (T-Cell-mediated) targeting of the IGF-system				
Second generation target Rx (multiple RTKs or ligands targeting MAbs)				
MAbs co-targeting of IGF1-IGF2 ligands				
m67 {bispecific scFv combining m610.27+m708.5}cc		Pharmacokinetic study in macaques		[154]
M708.5 {bispecific scFV to IGF-I/IGF-II}	Various tumor cell lines	In vitro anti-tumor activity		[155]
	Neuroblastoma	In-vivo xenograft antitumor		[156]
